# Feasibility of capsule endoscopy in elderly patients with obscure gastrointestinal bleeding. An up-to-date report

**DOI:** 10.1186/1471-2482-12-S1-S30

**Published:** 2012-11-15

**Authors:** G Orlando, IM Luppino, MA Lerose, R Gervasi, B Amato, G Silecchia, A Puzziello

**Affiliations:** 1 Gastroenterology and Endoscopy Unit, T. Campanella Oncological Foundation, Catanzaro, Italy; 2 General Surgery Unit, Dept of General Surgery, Geriatric and Endoscopy, University Federico II, Naples, Italy; 3 General Surgery Unit, Dept of Medical and Surgical Biotechnology and Sciences, University la Sapienza, Roma, Italy; 4 Endocrinosurgery Unit, Dept of Medical and Surgical Sciences, University Magna Graecia, Catanzaro, Italy

## Abstract

**Background:**

Anemia is the most common hematologic abnormality in older populations. Furthermore, iron deficiency anemia is common and merits investigation and treatment, as it usually results from chronic occult bleeding from the gastrointestinal tract. In view of a wide use of capsule endoscopy as a diagnostic procedure for occult gastrointestinal bleeding and of the growth of aging population, we performed a literature review about the feasibility of capsule endoscopy in the elderly.

**Methods:**

We conducted a literature search in the PubMed database in July 2012, and all English-language publications on capsule endoscopy in elderly patients since 2005 were retrieved. The potential original articles mainly focused on obscure gastrointestinal bleeding were all identified and full texts were obtained and reviewed for further hand data retrieving.

**Results:**

We retrieved only six papers based on different primary end-points. Four were retrospective non randomized studies and two were prospective non randomized studies. In the end 65, 70, 80 and 85 years were used as an age cut-off. All studies evaluate the diagnostic yield of capsule endoscopy in iron deficiency anemia. Only three studies assess the feasibility of capsule examination of the elderly.

**Conclusions:**

Iron deficiency anemia in the elderly with or without obscure gastrointestinal bleeding is the major indication for capsule endoscopy after a negative esophago-gastro-duodenoscopy and colonoscopy. It is safe and effective to identify a small bowel pathology without a great discomfort for the elderly. Inability to swallow the capsule, battery failure before capsule reaches the cecum, and capsule retention are some of the important problems associated with capsule endoscopy in elderly as well as in younger patients.

## Introduction

Anemia is the most common hematologic abnormality in older populations. Furthermore, iron deficiency anemia is common and merits investigation and treatment, as it usually results from chronic occult bleeding from the gastrointestinal tract. The incidence of gastrointestinal bleeding in patients over the age of 80 is high, over 350 cases per 100000 person-years [1]. Furthermore about 20% of the elderly have a negative upper and lower gastrointestinal endoscopy and two-thirds of theme have a lesion in the small bowel [2].

On the other hand, capsule endoscopy (CE) has developed an important role in the investigation of patients with obscure gastrointestinal bleeding (OGB) when upper endoscopy, push enteroscopy, colonoscopy, and barium contrast radiography of the small bowel are negative. CE is becoming so commonly used in practice and accepted by patients due to its noninvasive technique, compared with double-balloon endoscopy, that some authors also demonstrated its feasibility in patients who have undergone small-bowel resection for benign or malignant disease [3]. In view of a wide use of CE as a diagnostic procedure for OGB and of the growth of an aging population we performed a literature review on the utility and safety of CE in the elderly with obscure gastrointestinal bleeding. A second end-point of our study aims to confirm the spectrum of small bowel pathologies in this age group as a dominant cause of OGB.

Therefore, we performed this review of global literature to provide current state-of-the-art data on the number and type of CE-related publications, the indications, the diagnostic yield, the completion, and retention rates in evaluating OGB in the elderly.

## Material and methods

The literature search was conducted in the PubMed database in July 2012, and all English-language publications on CE since 2005 were retrieved. The search terms that we selected were ‘‘video capsule endoscopy OR capsule endoscopy OR wireless capsule endoscopy OR capsule endoscope OR video capsule endoscope AND elderly OR older adult’’ which were mainly based on the official thesaurus (MeSH). All initial search results were reviewed by title and abstracts. Then, the potential original articles mainly focusing on obscure gastrointestinal bleeding were all identified, and full texts were obtained and reviewed for further hand data retrieving.

We selected only six papers with different primary end-points. Four were retrospective non randomized studies and two were prospective non randomized studies. In the end 65, 70, 80 and 85 (oldest old) years were used as an age cut-off. All studies also evaluate the diagnostic yield of capsule endoscopy in iron deficiency anemia and more frequent CE findings in different age groups. Only three studies specifically assess the feasibility of capsule examination in the elderly with respect to bowel preparation, complete examination, patient’s compliance, transit time and capsule retention. Capsule retention was defined as a capsule endoscope remaining in the digestive tract for a minimum of 2 weeks or one that required directed intervention or therapy to aid its passage.

## Results

### Bowel preparation

Two studies [4,5] report a trend for poorer small bowel preparation in patient aged >65 years. This difference reached statistical significance in the proximal (p < 0.046) and in the distal (p < 0.048) half of CE small bowel transit time in respect to patients aged < 65 yr (5), and mainly in patients aged >80 years (Table 1) [4].

**Table 1:** Incomplete bowel preparation rate (modified from Tsibouris et al.)

	> 80 years	< 80 years	P value
**Incomplete bowel preparation**	17% (25/145)	3% (13/470)	< 0,0001

### Study discomfort

Only one study specifically reports a marked discomfort in age group (> 80 years) (p < 0,0001) with respect to the younger group. Furthermore, in the age group (> 80 years) 58% of patients found capsule endoscopy study very tiresome and difficult compared to 8% in the younger group (p< 0.0001) [4].

### Complete examination

Complete examination is defined as capsule passing through the ileocecal valve or into the colon in the images during its working time and capsule being excreted in 2 weeks, regardless of technical failure or poor small-bowel preparation. Two studies only report a rate of capsule not reaching the cecum of 6% and 7.5%. Two others randomized studies demonstrate that the percentage of patients in which the capsule reached the terminal ileum did not differ between aged and younger groups. (p_1_ = 0.78; p_2_ = 0.51) [4,5]. A prospective non randomized study also reports no difference in small bowel transit time (p > 0.78).

### Capsule endoscopy findings

The diagnostic yield of CE for the suspected bleeding source in all patients with OGB has been reported to range from 38% to 93%. The small bowel is the most likely source of bleeding in patients with continuing blood loss and negative upper and lower gastrointestinal endoscopies. No significant difference was found between the different age groups with regards to either the detection rate (p>0.05) or diagnostic rate (p>0.05), while there was significant difference (P < 10^− 3^) in CE study findings among different age groups. Angiodysplasia was the most prevalent finding (43.6%) in patient > 65 years, with a growing rate in patients > 80 years. Tsibouris et al. report in fact an 80% rate of angiodysplasia in aged group (median age 82.7 yr) with respect to the younger group (median age 63.4 yr) (p<0.0001).

## Discussion

In our study, we found no differences between the aged group and younger group with regard to the probability of completing the small bowel study and small bowel transit time. The p value for differences in the detection and diagnostic rates between the different age groups was non-significant, which suggests that CE examination has good detection and diagnostic capacity for OGB in individuals of various ages. The only relevant report was the poorer small bowel preparation in the elderly that could increase small bowel transit time and make video-capture worse. In the patient > 80 years with an iron deficiency anemia with or without OGB and negative upper endoscopy and colonoscopy the CE should have a high chance to reveal vascular anomalies. In fact diagnostic yield of CE in the evaluation of unexplained iron deficiency anemia progressively increases with advancing age and is highest among patients over 85 years of age (the oldest). This may be explained due to the fact that ASA, NSAIDs, and warfarin usage is more in older adults compared to younger patients.

## Conclusions

Since the first brief communication published in Nature in 2000 [6] introducing capsule endoscopy, CE has been so widely used in clinical practice that, although it is a useful tool for evaluating small bowel disease, some authors also demonstrated the utility and safety in patients who have undergone small-bowel resection for benign or malignant disease [3].

Nevertheless the most common indication remains the evaluation of OGB when upper endoscopy, push enteroscopy, colonoscopy, and barium contrast radiography of the small bowel are negative [7,8]. There is very little in the published literature on the use of CE in OGB in the elderly that data on contraindications and limitations are sparse. On the other hand as the population is rapidly ageing, it is expected that more elderly patients will undergo CE studies for various indications.

Although CE is a useful tool for evaluating small-bowel disease, it is impossible to view the entire small bowel in all patients because some capsules have not passed the ileo-cecal valve before battery exhaustion for various reasons. More randomized studies are necessary to examine if ageing affects either CE completion rate or the quality of bowel preparation, which are the two factors compromising the performance of CE and the small bowel transit time.

Ultimately, very few patients over the age of 80 have been included in capsule endoscopy studies, so it has been difficult to draw safe conclusions for this age group. Furthermore the selected studies took a different age cut off, using 65 years rather than 80 or 85 years (oldest old).

The main disadvantages of CE include capsule retention, false-negative results, and the lack of means for tissue proof or therapeutic intervention [9,10]. All patients in the aged group with suspected small bowel stenosis (previous history of incomplete ileus, prior abdominal operation) should receive a patency capsule before the capsule endoscopy examination as some authors suggest [4].

In conclusion, the use of CE can be considered another useful tool for the physician in the evaluation of OGB among the elderly, but other randomized studies are necessary to specifically evaluate in homogeneous age groups possible complications and contraindications.

## Competing interests

The authors declare that they have no competing interests.

## Authors' contributions

GO: conception and design, interpretation of data, given final approval of the version to be published; IML: acquisition of data, drafting the manuscript, given final approval of the version to be published; MAL: acquisition of data, drafting the manuscript, given final approval of the version to be published; RG: acquisition of data, drafting the manuscript, given final approval of the version to be published; BA: acquisition of data, drafting the manuscript, given final approval of the version to be published; GS: acquisition of data, interpretation of data, given final approval of the version to be published; AP: conception and design, critical revision, given final approval of the version to be published.
